# A cancer stem cell associated gene signature for predicting overall survival of hepatocellular carcinoma

**DOI:** 10.3389/fgene.2022.888601

**Published:** 2022-09-12

**Authors:** Xin-Yi Liang, Yue Zhang, Ya-Nan He, Xue-Yi Liu, Zhi-Hao Ding, Xiao-Dong Zhang, Ming-You Dong, Run-Lei Du

**Affiliations:** ^1^ Hubei Key Laboratory of Cell Homeostasis, College of Life Sciences, Wuhan University, Wuhan, China; ^2^ The Key Laboratory of Molecular Pathology (For Hepatobiliary Diseases) of Guangxi, Affiliated Hospital of Youjiang Medical University for Nationalities, Baise, China

**Keywords:** hepatocellular carcinoma, cancer stem cells, gene signature, immune, SGO2

## Abstract

Hepatocellular carcinoma (HCC) is the most prevalent type of primary liver cancer characterized by high mortality and morbidity rate. The lack of effective treatments and the high frequency of recurrence lead to poor prognosis of patients with HCC. Therefore, it is important to develop robust prediction tools for predicting the prognosis of HCC. Recent studies have shown that cancer stem cells (CSC) participate in HCC progression. The aim of this study was to explore the prognostic value of CSC-related genes and establish a prediction model based on data from The Cancer Genome Atlas (TCGA) database. In this study, 475 CSC-related genes were obtained from the Molecular Signature Database and 160 differentially expressed CSC-related genes in HCC patients were identified using the limma R package in the TCGA database. A total of 79 CSC-related genes were found to be associated with overall survival (OS). Using the least absolute shrinkage and selection operator (LASSO) and multivariate Cox regressions, a 3-gene signature (*RAB10*, *TCOF1,* and *PSMD14*) was constructed. Receiver operating characteristic (ROC) curves and Kaplan-Meier survival curves were constructed to test the prediction performance of the signature. Performance of the signature was validated using the International Cancer Genome Consortium (ICGC) dataset. In addition, immune feature and functional enrichment analyses were carried out to explore the underlying mechanisms. Moreover, a co-expression network was constructed using the weighted gene correlation network analysis (WGCNA) method to select genes significantly associated with risk scores in HCC in the TCGA dataset. The *SGO2* gene was found to be significantly associated with risk scores of HCC. *In vitro* experiments revealed that it can promote HCC cell proliferation. Therefore, *SGO2* may be a potential therapeutic target for HCC treatment. The constructed nomogram can help clinicians make decisions about HCC treatment.

## Introduction

Liver cancer, which is ranked sixth and third in terms of morbidity and mortality among all malignant tumors, has become a major public health issue worldwide (Sung et al., 2021). Of all subtypes of primary liver cancer, hepatocellular carcinoma (HCC) accounts for 80% of all liver cancer patients globally (Rumgay et al., 2022). Recent etiologic studies have shown that infection with HBV or HCV is the leading cause of HCC, and it mostly occurs in countries and regions with low economic development (Ghouri et al., 2017). In addition, alcohol addiction, metabolism-related liver disease, and dietary toxins, including aflatoxins and aristolochic acid, are common risk factors of HCC in some developed countries (El-Serag, 2012; Yang et al., 2019a). To date, there is no elaborate treatment strategy for HCC, which calls for studies to find a cure for this disease. Several molecularly targeted drugs have been approved for the treatment of HCC, but they only have moderate effects and are not effective in all patients (Huang et al., 2020). Although surgery, chemotherapy, and radiotherapy are widely used for the HCC treatment, the prognosis of patients is still poor, with a 5-years survival rate of only about 18% (Jemal et al., 2017). Prediction of HCC prognosis is hampered by the complex tumorigenesis mechanism, high degree of heterogeneity, and frequent recurrence.

Recent studies have demonstrated that liver cancer stem cells may be responsible for these malignant properties of HCC (Yamashita and Wang, 2013). Cancer stem cells (CSCs), also known as tumor-initiating cells, are a subpopulation of cells within the tumor that possess stem cell-like capacity. Lee et al. (2022) revealed that they are self-renewable and can differentiate into aggressive tumor cells to promote tumor growth. These cells may be the cause of tumor relapse and high heterogeneity. With the advancement in experimental technology, several biological markers of liver CSCs have been identified in HCC and were recently reviewed by (Tsui et al., 2020). Some markers, including CK19, ABCG2, CD44, and CD133, were found to be correlated with highly invasive features, and patients with elevated expression levels of these markers had worse prognosis and shorter survival time (Yang et al., 2010). Therefore, the CSCs-related gene signatures have the potential to become powerful predictors of prognosis for HCC patients.

In this study, the mRNA expression profiles and corresponding clinical data of HCC patients were retrieved from public databases and used to identify the key genes associated with liver CSCs. Consequently, a 3-gene signature model was constructed using The Cancer Genome Atlas (TCGA) cohort, and validated in the International Cancer Genome Consortium (ICGC) cohort. We confirmed that this gene signature was an independent predictor of overall survival (OS) and the underlying mechanisms were explored, with the overarching goal of providing a new strategy for accurately predicting the prognosis of HCC patients.

## Materials and methods

### Data collection

Gene expression profiles of RNA-sequencing data and corresponding clinical information of HCC patients were extracted from TCGA database (https://portal.gdc.cancer.gov/repository). Another dataset consisting of 231 samples was downloaded from the ICGC database. The TCGA-LIHC dataset was used as the training cohort and ICGC dataset as the validation cohort. As the data from both TCGA and ICGC are publicly available. Therefore, this study was exempted from approval by the local ethics committee. Patients with no follow up data from both TCGA and ICGC were excluded from the analysis. All read count values were normalized.

A total of 475 CSC genes from 109 CSC-related gene sets were obtained from a published Articles (Liang et al., 2020). The 109 CSC-related gene sets are provided in Supplementary Table S1.

### Construction and validation of a prognostic CSC-related gene signature

The “limma” R package was used to identify the differentially expressed genes (DEGs) between tumor and adjacent normal samples in the TCGA cohort, with false discovery rate (FDR) < 0.05 set as the cut-offs. Next, univariate Cox regression analysis of overall survival (OS) was performed to determine the prognostic value of CSC-related genes. 79 prognostic genes was obtained after examining the intersection between the two gene groups. Furthermore, to minimize overfitting, prognostic CSC-related genes were assessed by least absolute shrinkage and selection operator (LASSO) Cox proportional hazards regression using the “glmnet” R package. The value of penalty parameter (λ) corresponding to the lowest partial likelihood deviance was used to select the best model by 10-fold cross-validation. We got a list of genes with non-zero beta coefficients. Finally, a stepwise multivariate Cox regression analysis were used to establish a score system for calculating the survival risk for each HCC patient based on the expression level of each prognostic gene and its related regression coefficient. The following formula was used: risk score = sum (gene expression level * regression coefficient). Moreover, all patients were classified into high-risk or low-risk group according to the median value of risk score, which was used as the cut-off value. To observe the clustering conditions of the gene signature, principal component analysis (PCA), and t-SNE were performed using the “prcomp” function of the “stats” R package and the “Rtsne” R package, respectively. Time-dependent receiver operating characteristic (ROC) curves were generated using the “survivalROC” R package for evaluating the predictive capacity of the novel gene signature. The survival curves for different groups were analyzed with the Kaplan-Meier method with log-rank test. Finally, univariate and multivariate Cox regression analyses were performed to determine whether the CSC-related gene signature possessed the independent prognostic value. To verify the effectiveness of the model, the β value derived from the TCGA set was applied to the ICGC set, all patients come from ICGC were also classified into high-risk or low-risk group according to the same median value of risk score in TCGA set.

### Independence of the 3-CSC-related genes signature from clinical features of other TCGA-LIHC patients

Based on other clinical features (grade, age, TNM stage, and T stage) of TCGA-LIHC patients, univariate and multivariate Cox regression analyses were conducted to explore whether the prognostic model was an independent variable. To confirm the prognostic significance of the predictive model, TCGA-LIHC patients were divided into two groups according to different clinical characteristics. Patients were separately classified into the following subgroups: grade I/II, grade III/IV, stage I/II, stage III/IV, age <65, age ≥65, T1-T2, and T3-T4 subgroups. Survival outcome analysis was then performed to verify the independent prognostic significance of the gene signature in specific subgroups. The ideal cut-off value of the risk score was established using the surv_cutpoint function of “survminer” in R package.

### Immune infiltration analysis

Single-sample gene set enrichment analysis (ssGSEA) was performed using “ssGSEA” R package to calculate the infiltrating score of 16 immune cells and the activity of 13 immune-related pathways of patients in the high-risk and low-risk groups. Supplementary Table S2 shows the representative gene sets of immune cells and related pathways.

### Gene set variation analysis (GSVA)

Gene set variation analysis (GSVA) is a non-parametric and unsupervised GSE method which can calculate enrichment scores of predefined gene sets representing various biological processes in each sample (Hanzelmann et al., 2013). The GSVA was performed using the “clusterProfiler” R package to convert the gene expression profiles of patients in high-risk and low risk groups from TCGA and ICGC cohorts into the enrichment scores in biological signaling pathways or functions. The predefined pathway gene sets were obtained from the Kyoto Encyclopedia for Genes and Genomes (KEGG) database.

### Weighted gene correlation network analysis (WGCNA)

To identify the risk score-related hub genes, WGCNA was applied to HCC samples from the TCGA database using the “WGCNA” R package. This analysis method has two parts. One part classifies genes into different modules according to their expression patterns and the other identifies modules that are highly correlated with traits (Langfelder and Horvath, 2008). The WGCNA analysis was carried out on the identified DEGs in HCC tumor samples. First, a gene expression similarity matrix was constructed through calculating the Pearson correlation coefficient between any two genes. Next, a soft threshold of β = 8 was used to judge the similarity of two genes, followed by converting the similarity matrix into a weighted adjacency matrix. Finally, a topological overlap matrix (TOM) was applied to further measure the connectivity of genes in the co-expression network. Genes were clustered according to the value of 1-TOM. In addition, different modules were divided from the identified DEGs by building the dynamic pruning tree, with each module containing at least 30 genes. Similar modules were merged at the cut-off value of 0.25.

Co-expression network was constructed and genes were divided into modules, after which the relationships among modules and traits were determined, which included risk score and risk groups (low-risk = 0, high-risk = 1). Highly correlated modules were selected for further analyses. Gene ontology (GO) and KEGG analyses were then performed using the “clusterProfiler” R package to evaluate the biological functions of genes in the modules.

### Establishment and evaluation of a predictive nomogram

A nomogram was constructed based on gender, stage, grade, age, and risk score (Iasonos et al., 2008). The area under the ROC curve (AUC), 1-, 3-, and 5-years calibration curves, and decision curve analyses (DCA) (Vickers and Elkin, 2006) were then used to assess the nomogram’s prediction accuracy and discriminatory capacity.

### Cell culture

Human HCC cell lines Huh-7 and human kidney cells lines HEK293T were purchased from American Type Culture Collection. All cells were cultured in Dulbecco’s modified Eagle medium (HyClone, USA) supplemented with 10% fetal bovine serum (Gibco, USA) and 100U penicillin/streptomycin (HyClone, USA) at 37°C in a humidified thermal incubator with 5% CO_2_.

### CRISPR-Cas9-mediated SGO2 knockdown

Two optimal guide RNAs (CCA​GTC​TAT​TGG​CCG​CAG​AT and AAT​AGT​TCA​GAT​GTC​GAT​AT) targeting come from the different sites of the human SGO2 gene exon 6 were designed on the CRISPOR website (http://crispor.tefor.net/). Next, the gRNAs were cloned into pLentiCRISPRv2 (Addgene plasmid #52961). HEK293T cells (2x10^5^) were first seeded in 6-well plates and then transfected with pLentiCRISPRv2-gRNA plasmid or empty pLentiCRISPRv2 (2 µg) together with two lentiviral packaging plasmid psPAX2 (1 μg, Addgene plasmid #12260) and pMD2.G (1 μg, Addgene plasmid #12259) to generate lentivirus. After transfection for 48 h, the upper media containing lentivirus was collected and filtered using 0.45 µm membrane filters. On the other hand, Huh-7 cells were seeded in 6-well plates at a density of 2x10^5^ cells per well. After incubation for 24 h, the media were replaced with a mixture of two lentiviruses and fresh media (1:1:2) containing polybrene (10 μg/ml). The infected cells were selected by treatment with 1 μg/ml puromycine for 4 days until the death of control Huh-7 cells. Finally, the expression of *SGO2* was determined using quantitative real-time polymerase chain reaction (qRT-PCR).

### Cell proliferation and colony formation

Cell Counting Kit-8 (CCK-8, Dojindo Laboratories, Kumamoto, Japan) was used to explore the effect of *SGO2* knockdown on cell proliferation of HCC cells. Briefly, cells were first seeded into 96-well plates at a density of 1x10^3^ cells per well. Next, the CCK-8 solution (10 µl in 90 µl DMEM) was added to each well and incubated at 37°C for 1 h. Finally, the optical density of the medium was measured at a wavelength of 450 nm.

Colony formation experiment: Cells were placed in 6-well plates (1,000 cells per well). After 14 days of incubation, the colonies were fixed in 4% paraformaldehyde (Cat. 15,700, Electron Microscopy Sciences, USA) for 30 min, stained with 0.1% crystal violet (Beyotime, Beijing, China) for 15 min, and then washed it. Finally, colonies were photographed and counted using ImageJ software.

### Quantitative real-time PCR

Total RNA of wildtype and *SGO2* knockdown Huh-7 cells were extracted using TRIzol reagent (TAKARA, Japan). qRT-PCR was then performed with MonAmp™ ChemoHS qPCR Mix (Monad) in accordance with the manufacturer’s instructions. Relative expression of *SGO2* mRNA was normalized to *β-actin* mRNA (internal control) and calculated using the 2^-ΔΔCt^ method. The sequences of primer used were as follows: *SGO2*: 5'- GCC​CAG​TCT​ATT​GGC​CGC​AG -3' (forward) and 5'- TTC​AAT​CTT​TTC​CCC​AAT​AT -3' (reverse); *β-actin*: 5'- CAC​CAT​TGG​CAA​TGA​GCG​GTT​C -3' (forward) and 5'- AGG​TCT​TTG​CGG​ATG​TCC​ACG​T-3' (reverse).

### Statistical analyses

The students *t* test was used to evaluate gene expressions differences between normal and tumor tissues, while the chi-square test was used to compare proportional differences between normal and tumor tissues. The Kaplan Meier and log-rank tests were used for comparisons of OS for various clinical subgroup. Univariate and multivariate Cox regression analysis were used to identify independent predictors of OS. For data analysis, the R program (version 4.1.0) was utilized. Unless otherwise stated, *p* < 0.05 denotes significance.

## Results

### Identification of prognostic cancer stem cell (CSC)-related DEGs in TCGA cohort

**FIGURE 1:**
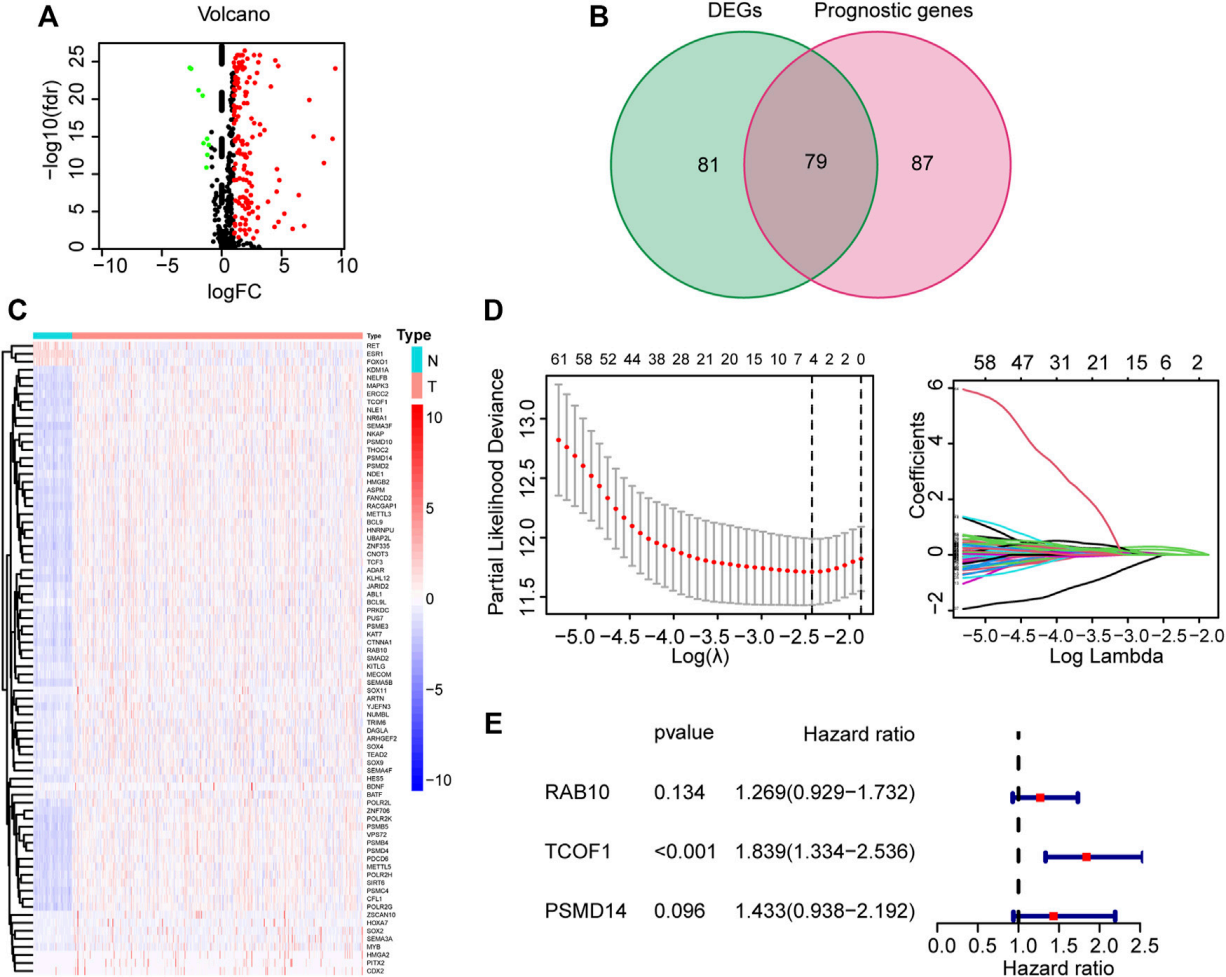
Identification and construction of CSC-related gene signature from TCGA corhot. **(A)** Volcano map of CSC-related DEGs in tumor and normal tissues from the TCGA dataset. **(B)** Venn plot used for selection of DEGs with prognostic value. **(C)** Heatmap illustrating differences in expression of 79 overlapping genes. **(D)** LASSO Cox regression analysis **(E)** Forest plot showing the relationship between the 3 genes and OS. CSC: cancer stem cell; DEG: Differentially expressed genes; LASSO: least absolute shrinkage and selection operator; OS: overall survival.

Supplementary Figure S1 shows the flow chart of the study. The detailed clinical characteristics of these patients come from TCGA are summarized in Table 1. A total of 160 CSC-related DEGs were identified between HCC cancer samples and adjacent normal samples (Supplementary Figures S2A,B). Among them, 79 DEGs were found to be associated with OS in the univariate Cox regression analysis (Figures 1A,B). Analysis of the heatmap plot showed that most of these prognostic DEGs were upregulated in the cancer samples (Figure 1C). GO analysis revealed that these CSC-related genes were mainly enriched in response to stem cell differentiation, mesenchyme development, mesenchymal cell differentiation, maintenance of stem cell population, and stem cell development (Supplementary Figure S2C). Moreover, KEGG pathway analysis revealed that these genes were correlated with the hippo signaling pathway, Wnt signaling pathway, TGF-beta pathway, and notch signaling pathway (Supplementary Figure S2D).

**TABLE 1:** Clinical characteristics of the HCC patients used in this study.

	TCGA cohort	ICGC cohort
No. of patients	365	231
Age (median, range)	61(16-90)	69(31-89)
Gender (%)		
Female	119(32.6%)	61(26.4%)
Male	246(67.4%	170(72.6%)
Grade (%)		
Grade 1	55(15.1%)	NA
Grade 2	175(47.9%)	NA
Grade 3	118(32.3%)	NA
Grade 4	12(3.3%)	NA
Unknown	5(1.4%)	NA
Stage (%)		
I	170(46.6%)	36(15.6%)
II	84(23.0%)	105(45.5%)
III	83(22.7%)	71(30.7%)
IV	4(1.1%)	19(8.2%)
unknown	24(6.6%)	0(0.0%)
Survival status OS days (median)	556	780
Survival status		
Dead	130	189
Alive	235	42

### Construction and evaluation of a CSC-related prognostic signature in the TCGA cohort

**FIGURE 2:**
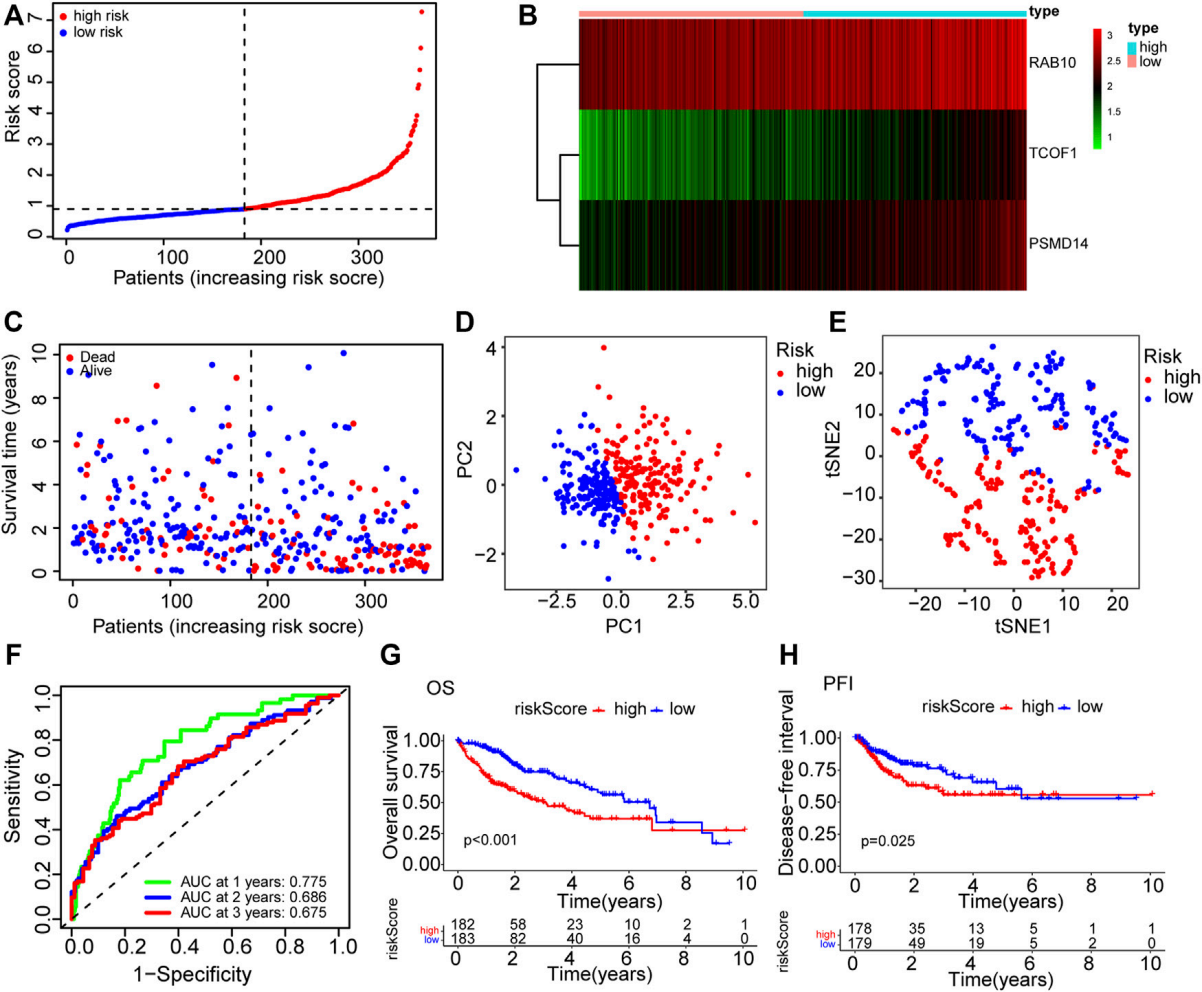
The prognosis prediction value of CSC-related gene signature in TCGA cohort. **(A)** The distribution of patients in high risk and low risk groups in the TCGA dataset. **(B)** Heatmap showing differences in expression levels of the 3 genes between the two risk groups. **(C)** The distribution of OS status and OS among various risk score groups. **(D)** PCA analysis of the TCGA dataset. **(E)** t-SNE analysis of the TCGA dataset. **(F)** AUC of time-dependent ROC curves for the risk score. **(G)** Kaplan-Meier survival curves showing the OS of patients in the two risk groups. **(H)** Kaplan-Meier survival curves showing the disease-free interval of patients in the two risk groups. AUC: area under the ROC curve; DFI: disease-free interval; OS: overall survival; PCA: Principal Component Analysis; ROC: Receiver Operating Characteristic.

To further filter the genes and reduce the risk of overfitting, LASSO Cox regression was employed. According to the multivariate Cox regression of OS, A 3-gene signature (*RAB10, TCOF1*, and *PSMD14*) was eventually identified based on the minimum value of λ (Figures 1D,E). Next, the risk score of each HCC patient in the TCGA dataset was calculated using the following formula: 0.238*expression level of *RAB10* + 0.609*expression level of *TCOF1*+0.360*expression level of *PSMD14*. Patients were stratified into two groups based on the risk score; patients with risk scores higher than the median value were classified in high-risk group (*n* = 182), whereas patients with risk scores lower than the median value were classified in low-risk group (*n* = 183) (Figure 2A). Results showed that the three genes were significantly upregulated in the high-risk group (Figure 2B). PCA and t-SNE analysis revealed that patients in the two groups were well distributed in different directions (Figures 2D,E). Further analysis showed that patients in the high risk score group had shorter the survival time and mortality risk (Figure 2C). Time-dependent ROC curves were generated to evaluate the prediction power of the risk score. Results demonstrated that the area under the curve (AUC) for 1-, 2-, and 3-years OS was 0.775, 0.686, and 0.675, respectively, indicating that the gene signature could predict the prognosis of HCC (Figure 2F). Analysis of Kaplan-Meier survival curves showed that patients in the high-risk group had poor OS and platinum-free interval (PFI) (*p* < 0.001; *p* = 0.025, Figures 2G,H).

### Validation of the prognostic model in the ICGC cohort

**FIGURE 3:**
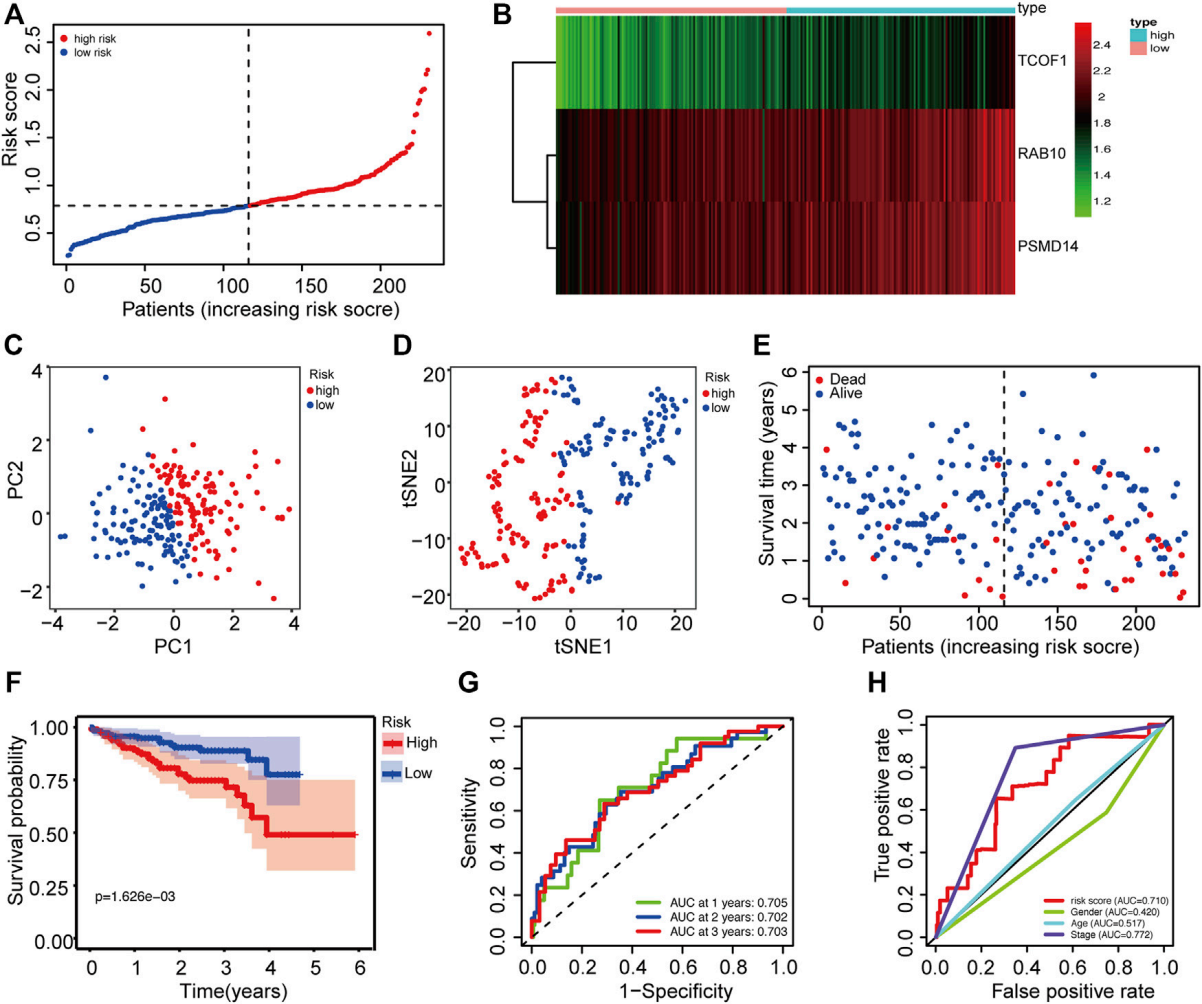
Validation of the prognostic prediction accuracy of the signature in the ICGC cohort. **(A)** The distribution of patients between high risk and low risk groups in the ICGC dataset. **(B)** Heatmap showing differences in expression levels of 3 genes between the two risk groups. **(C)** PCA analysis for the ICGC dataset. **(D)** t-SNE analysis for the ICGC dataset. **(E)** The distribution of OS status and OS among various risk score groups. **(F)** Kaplan-Meier survival curves for the OS of patients in the two risk groups. **(G)** AUC of time-dependent ROC curves for the risk scores. **(H)** AUC of ROC curves for the risk score and various clinical characteristics. AUC: area under the ROC curve; DFI: disease-free interval; OS: overall survival; PCA: Principal Component Analysis; ROC: Receiver Operating Characteristic.

The dataset downloaded from the ICGC was used to verify the generality of the novel CSC-related prognostic model. The risk score was used to divide patients from the ICGC dataset into high-risk groups and low-risk groups (Figure 3A). Similarly, the three prognostic risk genes were upregulated in the high-risk group (Figure 3B). PCA and t-SNE analyses indicated that patients in the different risk groups were distributed in two discrete sections (Figures 3C,D). Results of the scatterplot and Kaplan-Meier survival curves showed that patients with high-risk scores had short survival time and high mortality rate (Figures 3E,F), consistent with findings in the TCGA cohort. The AUC of time-dependent ROC ranged from 0.705 to 0.703 for 3 years (Figure 3G), indicating that the gene signature could predict the prognosis of patients in the ICGC cohort. Moreover, the risk score showed better prediction than most the clinical characteristics such as gender and age (Figure 3H).

### Independent prognostic value of the 3-CSC-related genes signature

**FIGURE 4:**
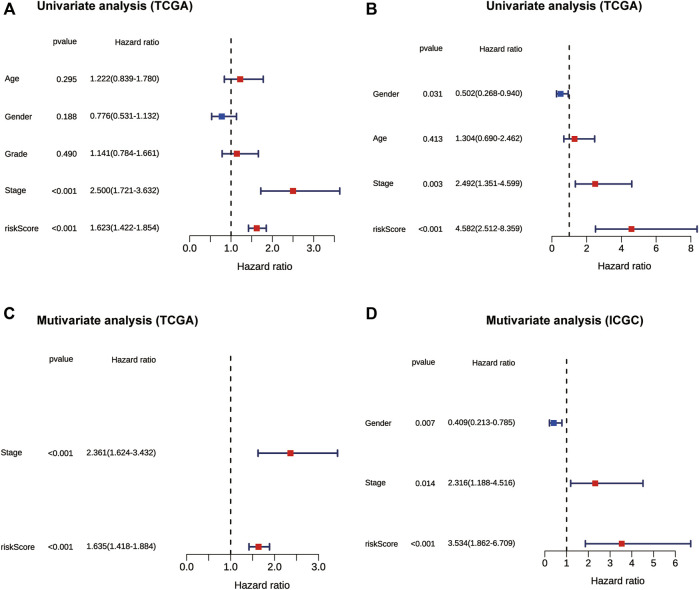
The forest plot showing results of univariate and multivariate Cox regression analyses of OS in the TCGA **(A,C)** and ICGC cohort **(B,D)**. OS: overall survival.

To further evaluate the prognostic value of the risk score, univariate and multivariate Cox regression analyses were applied among risk score and some clinicopathological characteristics in both TCGA and ICGC cohorts. The univariate Cox regression analysis results showed that the risk score (*p* < 0.001, HR = 1.623, 95% CI = 1.422-1.854) and tumor stage (*p* < 0.001, HR = 2.500, 95% CI = 1.721-3.632) were significantly associated with OS in the TCGA cohort (Figure 4A). In the multivariate Cox regression analysis of the two variables, the risk score (*p* < 0.001, HR = 1.635, 95% CI = 1.418-1.884) and tumor stage (*p* < 0.001, HR = 2.361, 95% CI = 1.624-3.432) were independent prognostic factor in the TCGA dataset (Figure 4C). The risk score was also an independent prognostic factor in the ICGC cohort (*p* < 0.001, HR = 3.534, 95% CI = 1.862-6.709) (Figure 4B, Figure 4D). Collectively, these results suggest that the constructed risk score was an independent prognostic predictor of OS.

To assess the prognostic value of the model in various clinic-pathological subgroups, clinical variables and samples were randomized into two subgroups in term of TNM stage, age, grade, and T stage. The obtained results indicated that the 3-CSC-related genes signature was significantly correlated with the survival of patients in different clinic-pathological subgroups in the TCGA-LIHC cohort (Supplementary Figure S3).

### The correlation between immune status and risk score

**FIGURE 5:**
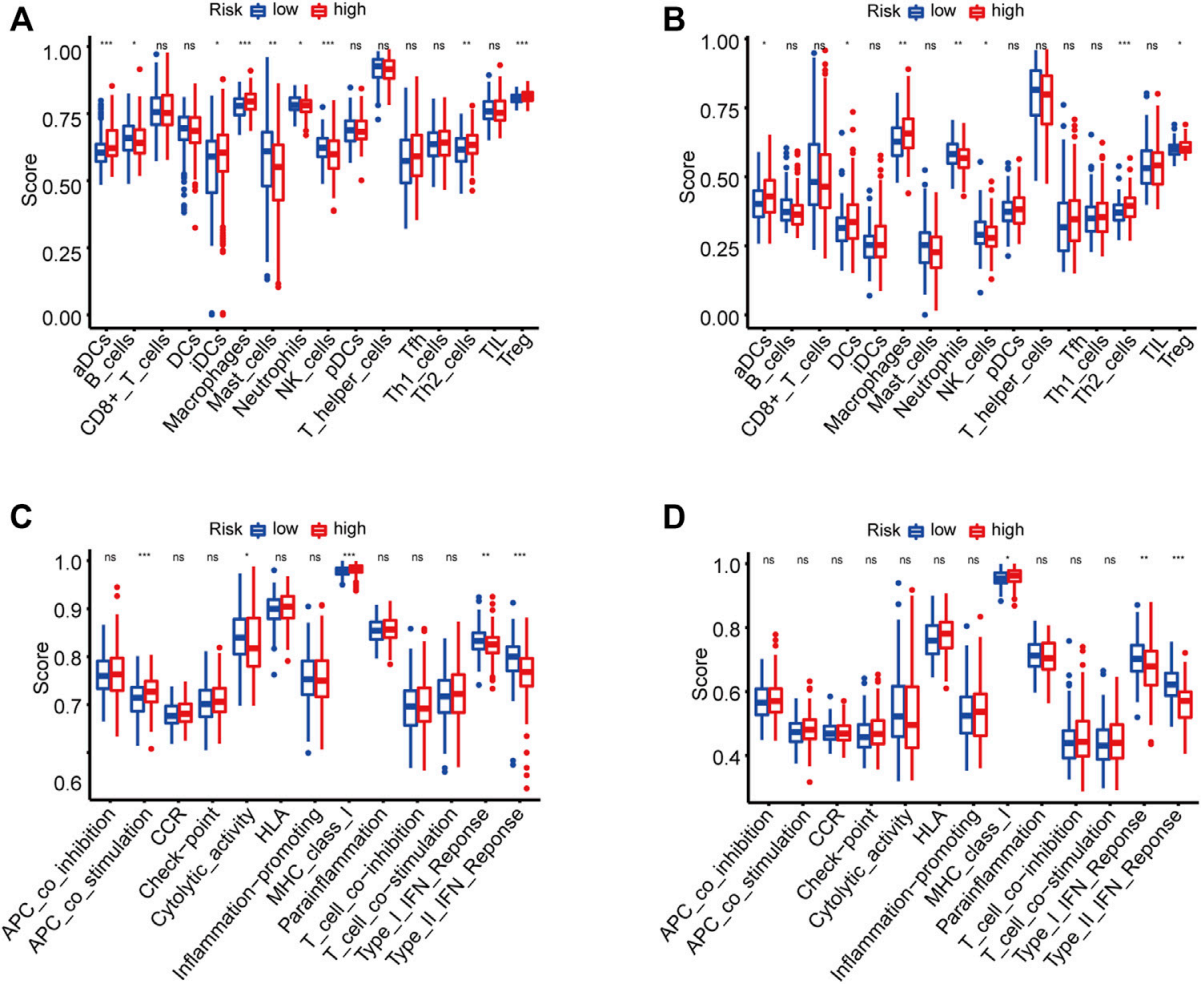
The enrichment scores of 16 types of immune infiltrating cells and 13 immune-related functions in the TCGA **(A,C)** and ICGC **(B,D)** cohorts. *p < 0.05, **p < 0.01, ***p < 0.001.

HCC patients were classified into high-risk and low-risk groups based on the risk score. It was evident that patients with high-risk scores were more likely to have poor prognosis, which has been confirmed above. However, the correlation between poor prognosis and high-risk score was not clear. We suspected that there might be a difference in immune status between the two risk groups. To this effect, the ssGSEA method was applied to all HCC samples in the high-risk and low-risk groups to evaluate immune cells infiltration and the activities of immune-related pathways. Figure 5A and Figure 5B show the enrichment scores of 16 types of infiltrating immune cells. In TCGA and ICGC cohorts, the scores of aDCs, macrophages, Th2 cells, and Tregs were significantly upregulated in the high-risk score group, whereas the scores of neutrophils and NK cells were lower than those in the low-risk group (*p* < 0.05). Among the screened immune-related functions or pathways, the expression of MHC class I was increased in the high-risk score group. In contrast, the activities of type I IFN response and type II IFN response were inhibited in the high-risk score group (*p* < 0.05, Figures 5C,D).

### Identification of biological functions associated with the risk score

**FIGURE 6:**
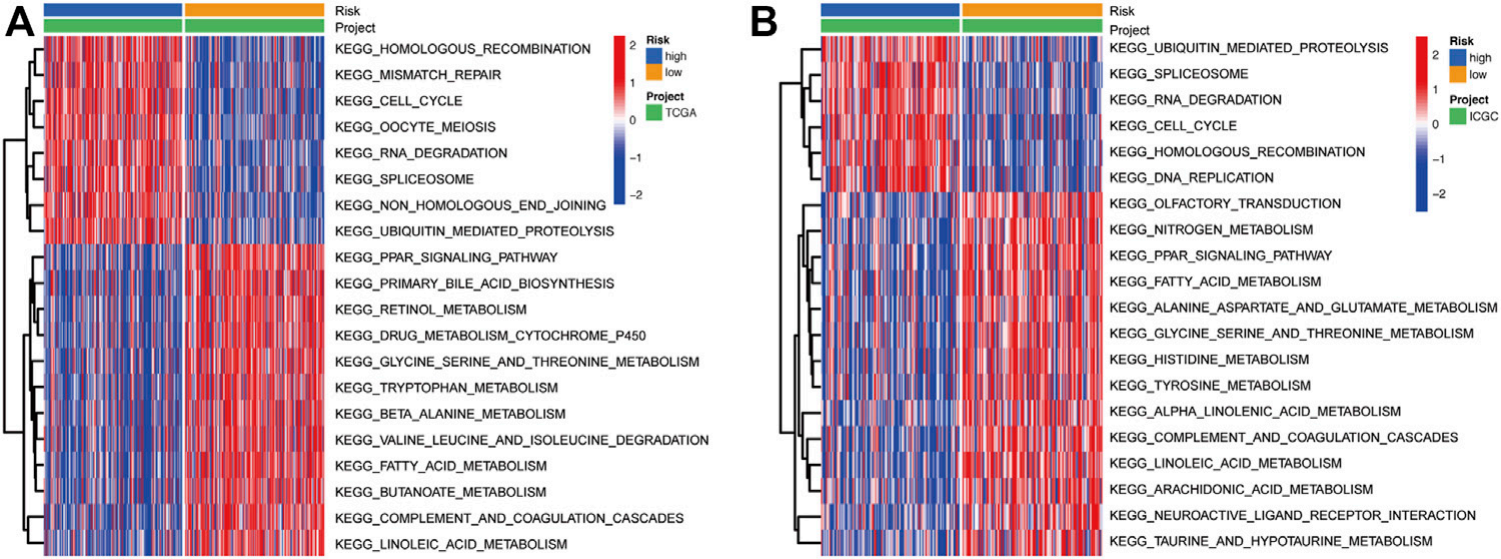
Heatmap showing the KEGG pathways enriched in high- and low-risk groups in the TCGA **(A)** and ICGC **(B)** cohorts. KEGG: Kyoto Encyclopedia of Genes and Genomes.

To further explore the differences in the activities of various biological signaling pathways between high-risk and low-risk groups, GSVA enrichment analysis was performed for each sample from the TCGA and ICGC cohorts. Results showed that the common pathways and functions enriched in samples from high-risk groups in both TCGA and ICGC cohorts were homologous recombination, cell cycle, RNA degradation, splicesome, and ubiquitin mediated proteolysis (Figures 6A,B). On the other hand, the activities of various metabolism-related pathways in the low-risk groups were substantially higher than those in the high-risk groups, including glycine-serine and threonine metabolism, fatty acid metabolism, linoleic acid metabolism, and the PPAR signaling pathway. In addition, one immune-related pathway, complement and coagulation cascades, was enriched in the low-risk groups (Figures 6A,B).

### Hub genes associated with high-risk in HCC patients

**FIGURE 7:**
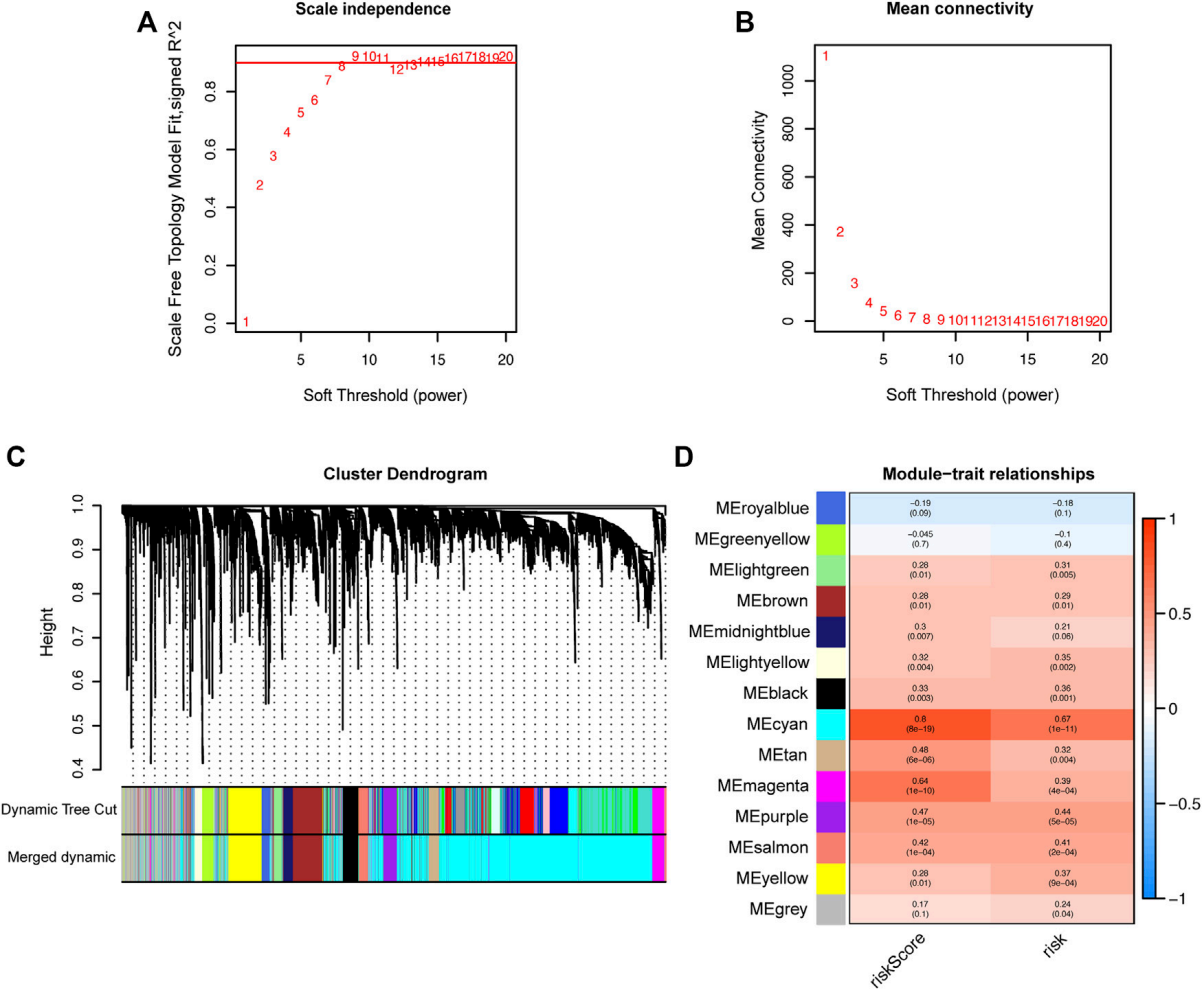
Identification of hub genes related to the risk score by WGCNA. **(A)** Analysis of the optimal soft-thresholding power based on scale-free network. **(B)** Mean connectivity analysis of soft-thresholding powers. **(C)** Cluster dendrogram of co-expressed genes. Each branch represents a module and is given a unique color. **(D)** Heatmap showing the correlation coefficients (upper row) and significance (*p* values showed in the brackets) between modules and risk score or risk groups. WGCNA: Weighted Gene Co-expression Network analysis.

To screen out the hub genes responsible for the high-risk of poor prognosis in HCC, WGCNA was performed on the DEGs between tumor and normal tissues identified from TCGA database. A co-expression network of 5,301 genes was built and 14 modules were divided from it through setting the optimal soft-thresholding power at 8 (scale free R^2^ = 0.85, Figures 7A–C). The correlation coefficients between each module and risk score or risk groups (high-risk = 1; low-risk = 0) were calculated and presented in a heatmap. The results suggested that the cyan module was the most closely and positively associated with risk score and the high-risk group, with correlation coefficients of 0.8 and 0.67, respectively (*p* < 0.001, Figure 7D). Therefore, the cyan module was selected for further analysis. GO and KEGG enrichment analyses were conducted on the 2,815 genes in the cyan module to reveal the functional link between key genes. The top 10 GO terms and KEGG pathways enriched from the genes of cyan module are shown in Supplementary Figure S4.

**FIGURE 8:**
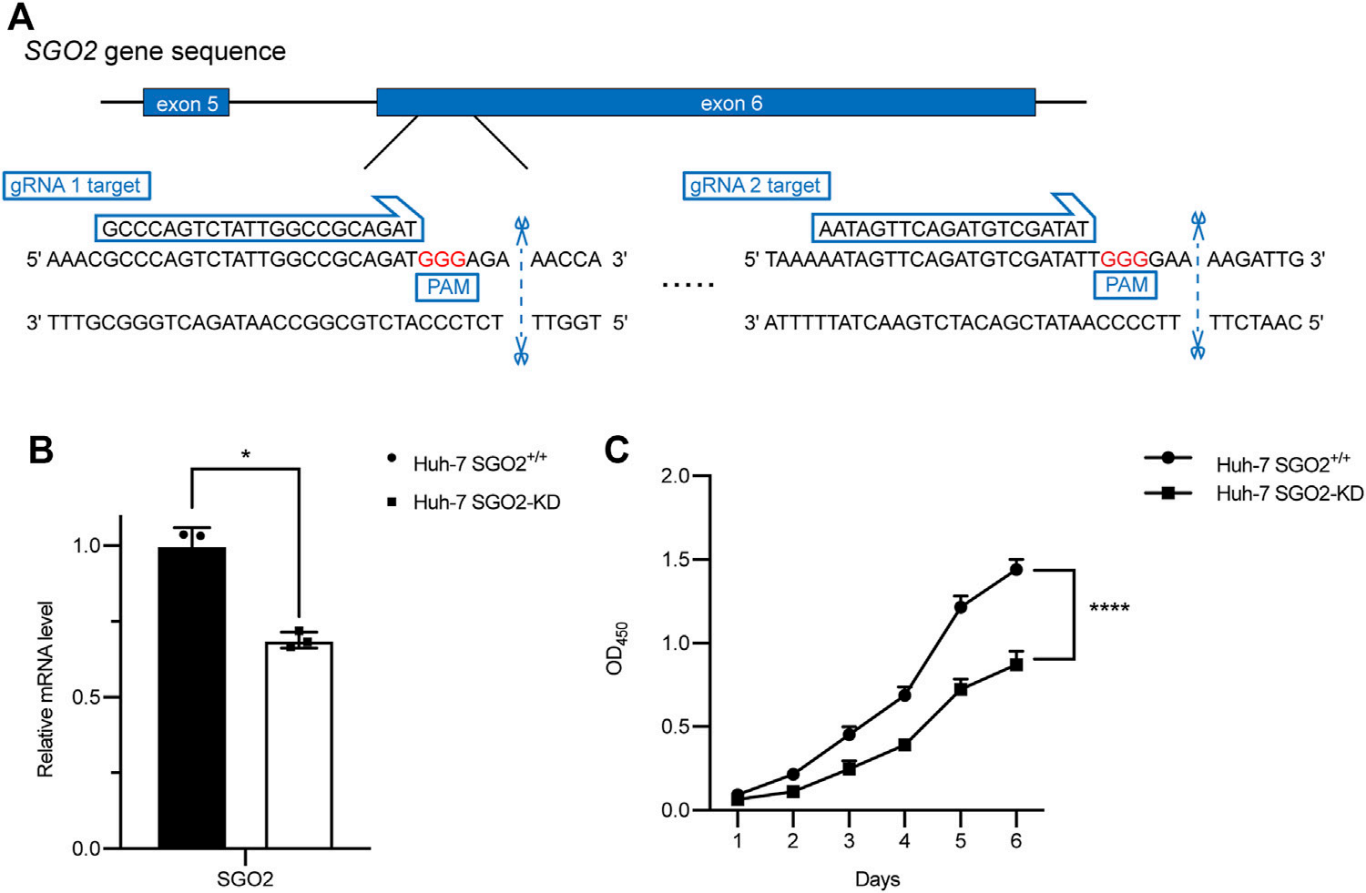
CRISPR-Cas9 mediated SGO2 knockdown inhibits the proliferation of HCC cells. **(A)** Two specific gRNAs targeting the gene sequence of *SGO2*. **(B)** Confirmation of expression level of SGO2 *mRNA* in Huh-7 SGO2 knockdown cells and control cells by Quantitative real-time PCR assay. **(C)** The cell proliferation status, evaluated daily for 6 days using the CCK-8 assay (*n* = 6). CCK-8: Cell Counting Kit-8; HCC: Hepatic cell carcinoma; PCR: Polymerase Chain Reaction.

Genes in the module were ranked by the scores and *p* values of gene significance (GS) and module membership (MM). Notably, the top 10 genes are listed in Table 2. Given that the mechanism of the number 1 gene (*BUB1B*) in HCC has already been elucidated (Qiu et al., 2020), we chose the number 2 key gene (*SGO2*) for subsequent analyses. Notably, *in vitro* experiments were conducted to further investigate the biological functions of *SGO2* in HCC. Specifically, the expression level of *SGO2* was knocked down in Huh-7 cells by CRISPR/Cas 9 system as illustrated in Figure 8A. The success of *SGO2* knockdown was confirmed using qRT-PCR analysis (Figure 8B). The CCK-8 assay results showed that decreased expression of *SGO2* had a significant inhibitory effect on the proliferation of Huh-7 cells (Figure 8C).Clone formation experiments showed, Huh-7 cells with knockdown of *SGO2* showed worse ability to form colonies.The difference was statistically significant (*p* < 0.05), as shown in Supplementary Figure S5. The results showed that *SGO2* knockdown effectively inhibited the clone formation ability of HCC cells.

**TABLE 2:** Genes in the cyan module ranked by scores and *p* values of MM and GS.

Gene	MM	Correlation coefficients and *p*-values	
		Risk score	Risk group
BUB1B	0.8407 (3.29E-22)	0.8814 (8.60E-27)	0.5789 (2.28E-08)
SGO2	0.9126 (1.24E-31)	0.8558 (9.66E-24)	0.6264 (6.65E-10)
STIP1	0.7749 (5.29E-17)	0.8464 (9.16E-23)	0.7098 (2.43E-13)
NCAPG	0.7764 (4.21E-17)	0.8447 (1.34E-22)	0.5237 (7.33E-07)
PRC1	0.7758 (4.61E-17)	0.8339 (1.44E-21)	0.5418 (2.51E-07)
HJURP	0.8376 (6.54E-22)	0.8315 (2.40E-21)	0.5658 (5.53E-08)
SPC25	0.7419 (5.22E-15)	0.8312 (2.52E-21)	0.5789 (2.28E-08)
KPNB1	0.8374 (6.82E-22)	0.8294 (3.69E-21)	0.5746 (3.08E-08)
SPATS2	0.8872 (1.39E-27)	0.8278 (5.16E-21)	0.6553 (5.63E-11)
KIF23	0.8164 (4.77E-20)	0.8263 (6.89E-21)	0.5478 (1.74E-07)

### Establishment and validation of the predictive nomogram

**FIGURE 9:**
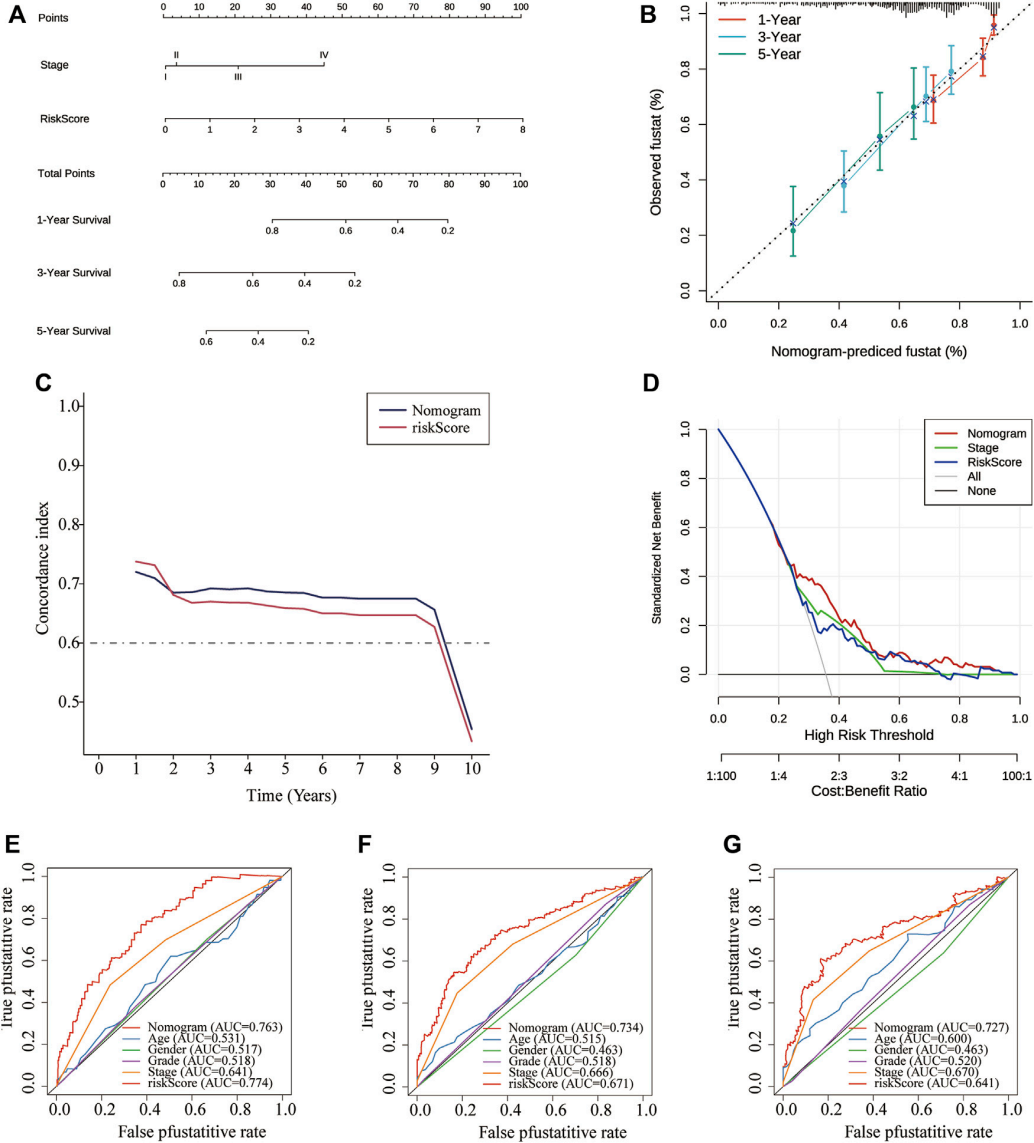
Construction of a nomogram based on independent prognostic factors for predicting OS in TCGA cohort. **(A)** The prediction performance of the nomogram for the OS at 1-, 3- and 5-years. **(B)** Calibration curves for the predicted 1-, 3-, and 5-years OS. **(D)** DCA curve. **(C)** Concordance index demonstrating the concordance measure of the predictor with patient survival. **(E–G)** ROC curves for the predicted 1-, 3-, and 5-years OS. DCA: decision curve analyses; LIHC: Liver hepatocellular carcinoma; OS: overall survival; ROC: Receiver Operating Characteristic.

To make the prognostic signature more convenient for clinical application, we constructed a nomogram and tested its capacity to predict OS on the TCGA-LIHC cohort at 1-, 3-, and 5-years based on risk scores, and other signifcant independent risk factors (Figure 9A). The 1-, 3-, and 5-years OS calibration curves of the TCGA-LIHC data revealed that the nomogram had good predictive discrimination and accuracy (Figure 9B). Moreover,the nomogram had a higher consistency index compared to other clinical markers and the risk score (Figure 9C). In addition, Comparison of the net benefits of various models, such as none, risk score, all, nomogram, and clinical indicators revealed that the nomogram had a higher net income and a wider threshold probability (Figure 9D). The analysis results revealed that the nomogram had a better prognostic ROC value compared to the 3-CSC-related-genes signature and other four clinical indicators, and it could predict OS for 1-, 3-, and 5-years (Figures 9E–G).Finally, the predictive value of nomogram was further validated in the ICGC dataset (Supplementary Figure S6). Altogether, results of ROC, DCA, calibration curve, and C index analyses indicated that the nomogram had better clinical benefits than risk scores based on the four 3-genes signatures alone.

## Discussion

Herein, 456 cancer stem cell-related genes were identified in HCC tumor samples. The correlation of these genes with clinical outcomes was explored. Among them, three genes (*RAB10*, *TCOF1,* and *PSMD14*) were found to be significantly upregulated in tumor samples, and univariate Cox regression showed that they were also significantly associated with overall survival. Consequently, the three genes were used to construct a novel prognosis prediction model. The effectiveness of the model as a prognosis predictor was then confirmed using internal and external validation cohorts, with results showing that it was an independent variable of HCC prognosis.

Previous studies reported the biological functions of the three genes in cancers. Consistent with our results, the expression level of these genes was upregulated in human HCC (He et al., 2002; Lv et al., 2020; Wu et al., 2022). *RAB10* is a member of the RAS superfamily. It encodes a protein that functions as small GTPase and plays a crucial role in intercellular vesicular trafficking (Pereira-Leal and Seabra, 2000; Chua and Tang, 2018). Some studies have revealed that *RAB10* knockdown significantly suppressed proliferation of HCC cells both *in vitro* and in nude mice xenografts, suggesting that *RAB10* was involved in tumorigenesis (Wang et al., 2017). Furthermore, *RAB10* silencing could induce cell cycle arrest and apoptosis in HCC cells, thereby affecting a number of cellular signaling pathways (Wang et al., 2017). *TCOF1* encodes the protein named treacle, a nucleolar factor that can regulate the transcription of not only ribosomal DNA but even of DNA elsewhere in the genome (Valdez et al., 2004). It has been found to activate the transcription of oncogenes and increase ribosomal production in HCC cells, thereby promoting tumor growth (Wu et al., 2022). Wu *et al.* (2022) reported that expression of *TCOF1* can alter the immune microenvironment of HCC and induce the antitumor immune cell infiltration, which indirectly facilitates HCC progression. Furthermore, although not reported in HCC, the tumor-initiating capacity and self-renewal ability of triple-negative breast cancer cells were affected by *TCOF1* depletion, which indicates its significance in maintaining CSC stemness (Hu et al., 2022). *PSMD14* is a subunit of the 19S regulatory cap of the 26S proteasome that mediates substrate deubiquitination, a deubiquitinase belonging to the JAMM domain metalloprotease family. It has recently been identified as an oncogene, and has been shown to be associated with multiple solid tumors and poor prognosis (Luo et al., 2017; Song et al., 2017). Moreover, *PSMD14* has been found to interact with various signaling pathways in HCC, such as stabilizing E2F1 to hyperactivate its downstream pro-survival signaling thereby promoting cell proliferation (Wang et al., 2015); deubiquitinating TGF-β receptors (TGFBR1 and TGFBR2) and CAV1 to facilitate tumor metastasis (Wang et al., 2019), and protecting GRB2 from degradation to regulate HCC progression (Lv et al., 2020). In summary, these three genes modulate HCC tumorigenesis, and influence cancer progression and prognosis through complex mechanisms. Therefore, they can be use as risk factors for evaluating clinical outcomes in HCC patients.

In this study, patients were classified into two groups based on the 3-gene signature: high-risk or low-risk group. Experiments were conducted based to uncover the potential relationships between CSC and poor prognosis in HCC from a holistic point of view. Considering that most HCC cases were driven by chronic liver inflammation, the patients’ immune status was of concern. GSVA results showed that that homologous recombination, cell cycle, RNA degradation, splicesome, and ubiquitin mediated proteolysis were positively enriched in TCGA and the ICGC cohort, which suggested that the dysregulation of these pathways was closely related to HCC development. The ssGSEA analysis found that high-risk patients tended to have higher infiltration levels of macrophages and Treg cells in TCGA and the ICGC cohort. Previous studies have demonstrated that increased tumor-associated macrophages (Zhang et al., 2019; Zhou et al., 2016) or Treg cells (Fu et al., 2007; Zhou et al., 2016) are related to poor prognosis in HCC patients due to their role in immune invasion. Of note, these immune cells have previously been identified as immunosuppressive cells in the tumor microenvironment that can inactivate anti-tumor immunity and help tumor cells escape immune attack, thereby promoting tumor growth. They have also been reported to be associated with poor prognosis of HCC patients (Dong et al., 2016; Fu et al., 2007; Lu et al., 2019). Moreover, some anti-tumor immune responses were impaired in the high-risk group, including decreased neutrophils and NK cells as well as the activity of type I and type II IFN response. In line with our results, a recent study by Dai et al. (2021) found that CSCs were involved in immune evasion, suggesting that they can be used as immunotherapeutic targets for HCC. Therefore, one reasonable explanation for the poor prognosis of patients in the high-risk group is the immunosuppressive microenvironment created by cancer stem cells. Furthermore, GSVA results suggested that homologous recombination, cell cycle, RNA degradation, splicesome, and ubiquitin mediated proteolysis were positively correlated with risk score. In addition, the activity of many metabolism pathways, including glycine-serine and threonine metabolism, fatty acid metabolism, linoleic acid metabolism, and the PPAR signaling pathway, was inhibited in the high-risk group. These results suggest that dysfunction of these signaling pathways may contribute to cancer progression, which is worthwhile for future studies and provides new insights into the molecular mechanisms of tumorigenesis and the search for a HCC cure.

Finally, to determine key genes significantly associated with HCC prognosis, we performed WGCNA using DEGs identified from the TCGA dataset, with risk scores and risk groups as the traits. A gene module was identified from the constructed co-expression network. *BUB1B*, the first gene in the module, has been reported to promote HCC progression by activating the mTORC1 signaling pathway (Qiu et al., 2020). It has been found to have highly expressed in tumor tissues and HCC cell lines, and *BUB1B* knockdown significantly inhibited the proliferation, migration, and invasion of HCC cells (Qiu et al., 2020). Moreover, several bioinformatics analyses have considered it as a prognostic marker and potential therapeutic target for HCC (Fu et al., 2021; Yang et al., 2019b; Zhou et al., 2019; Zhuang et al., 2018). Therefore, we hypothesized that the second gene in the same module, *SGO2*, may play a similar oncogenic role as *BUB1*, although its biological function in HCC remains poorly understood. Results obtained after conducting the *in vitro* experiment suggested that *SGO2* plays a critical role in HCC cell proliferation, and knockdown of *SGO2* largely suppressed cell growth. However, further mechanistic studies should be performed to investigate the oncogenic role of *SGO2*, and explore its value as a new therapeutic target and prognostic marker of HCC.

In summary, this study identified three cancer stem cell related genes that were associated with poor prognosis in HCC patients. The three genes were used to construct a novel model for accurate and independent prediction of clinical outcome of HCC patients. Therefore, this model can be widely used to predict the prognosis of HCC patients, and provides valuable insights for improving patient outcomes. Moreover, this study demonstrates for the first time the tumor-promoting role of *SGO2* in HCC using cellular experiments. Collectively, our results provide a fundamental contribution to elucidating the pathogenesis of HCC as well as the search for new therapeutic targets in the future.

## Data Availability

The datasets presented in this study can be found in online repositories. The names of the repository/repositories and accession number(s) can be found in the article/Supplementary Material.
