# Supplementary figures and images for: A cancer stem cell associated gene signature for predicting overall survival of hepatocellular carcinoma
Source: Front Genet. 2022 Sep 12;13:888601. doi: 10.3389/fgene.2022.888601 (PMC9511042; doi:10.3389/fgene.2022.888601)

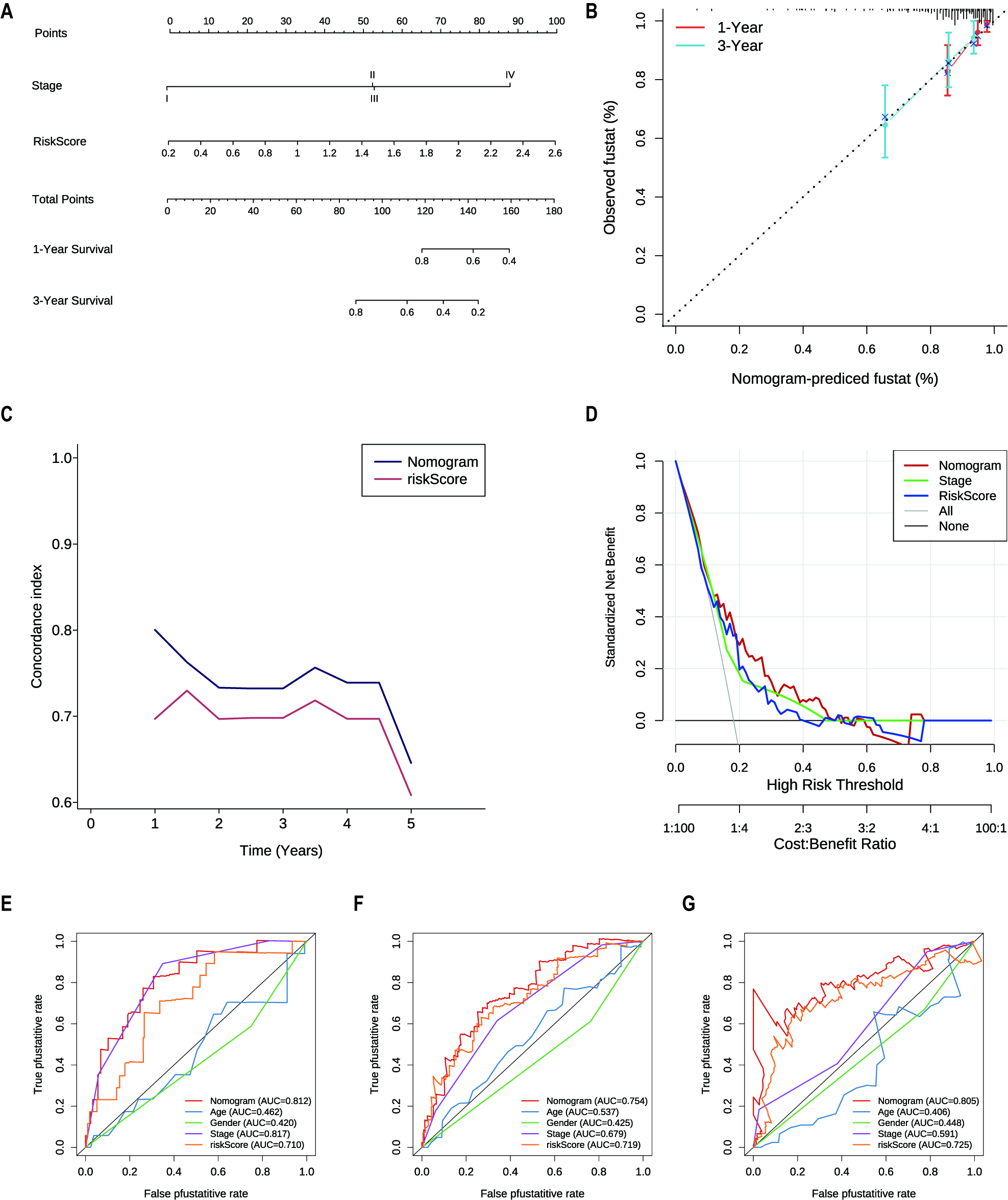

Supplement: Supplementary file 1 [file Image6.TIF]

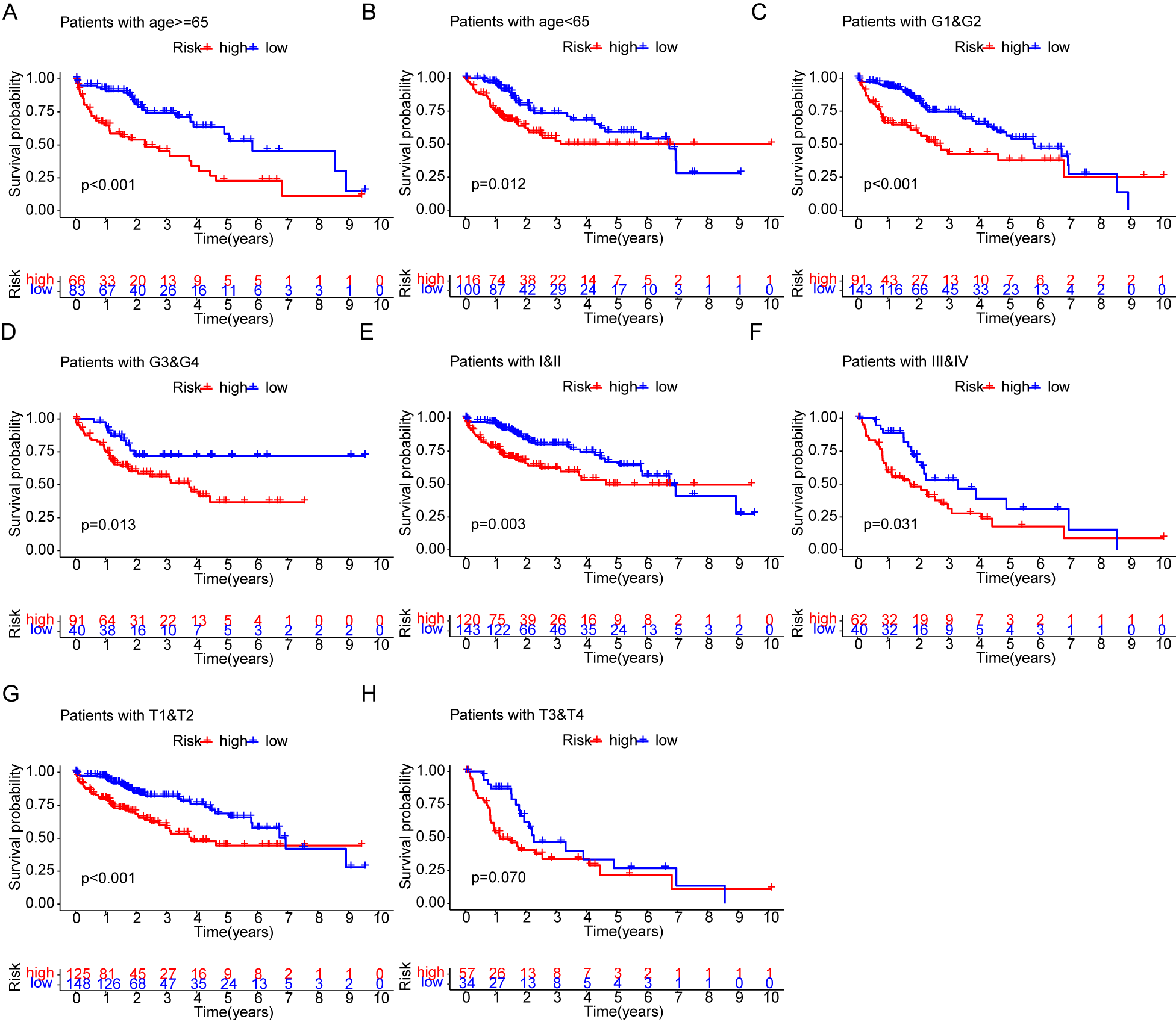

Supplement: Supplementary file 2 [file Image3.TIF]

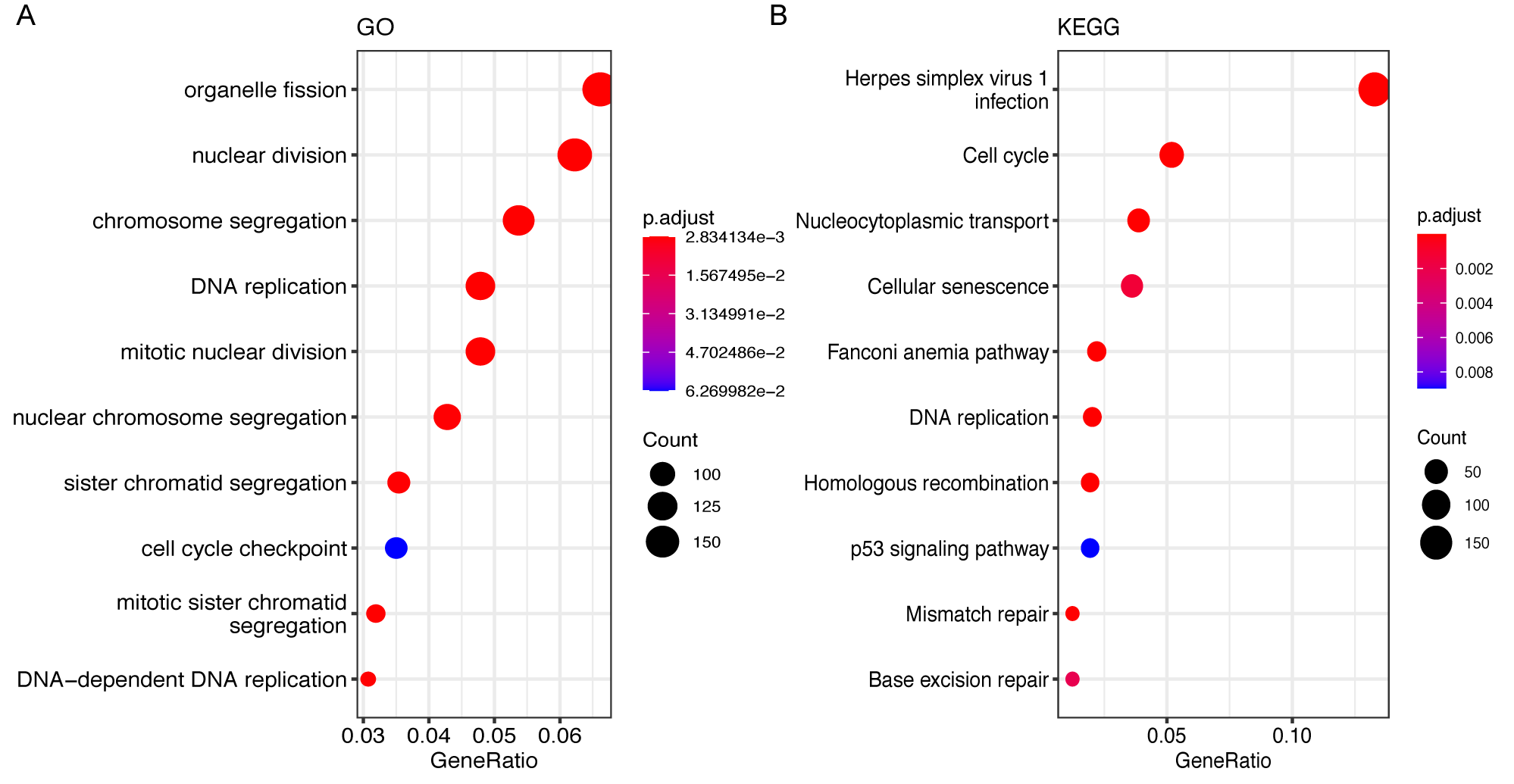

Supplement: Supplementary file 3 [file Image4.TIF]

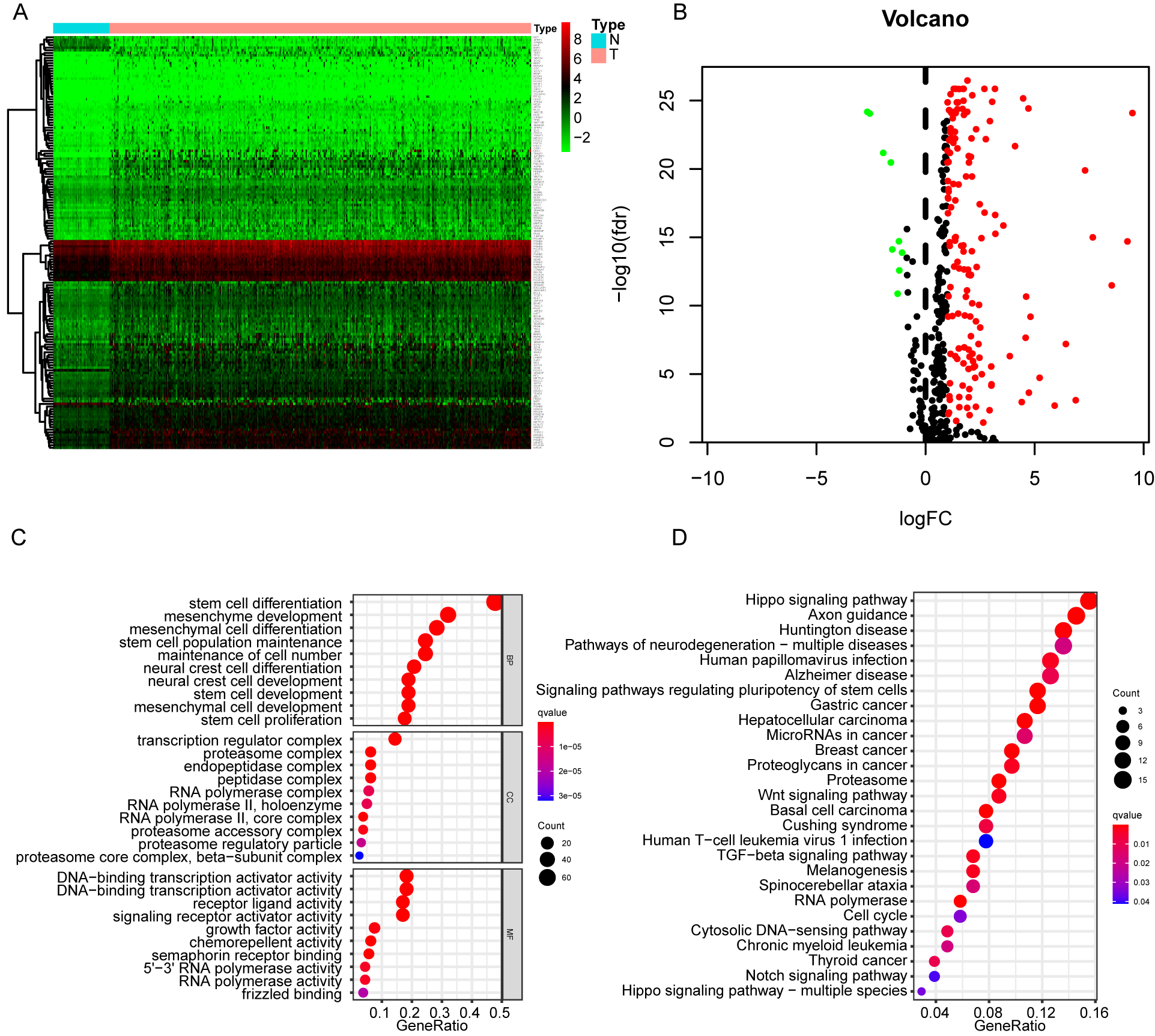

Supplement: Supplementary file 4 [file Image2.TIF]

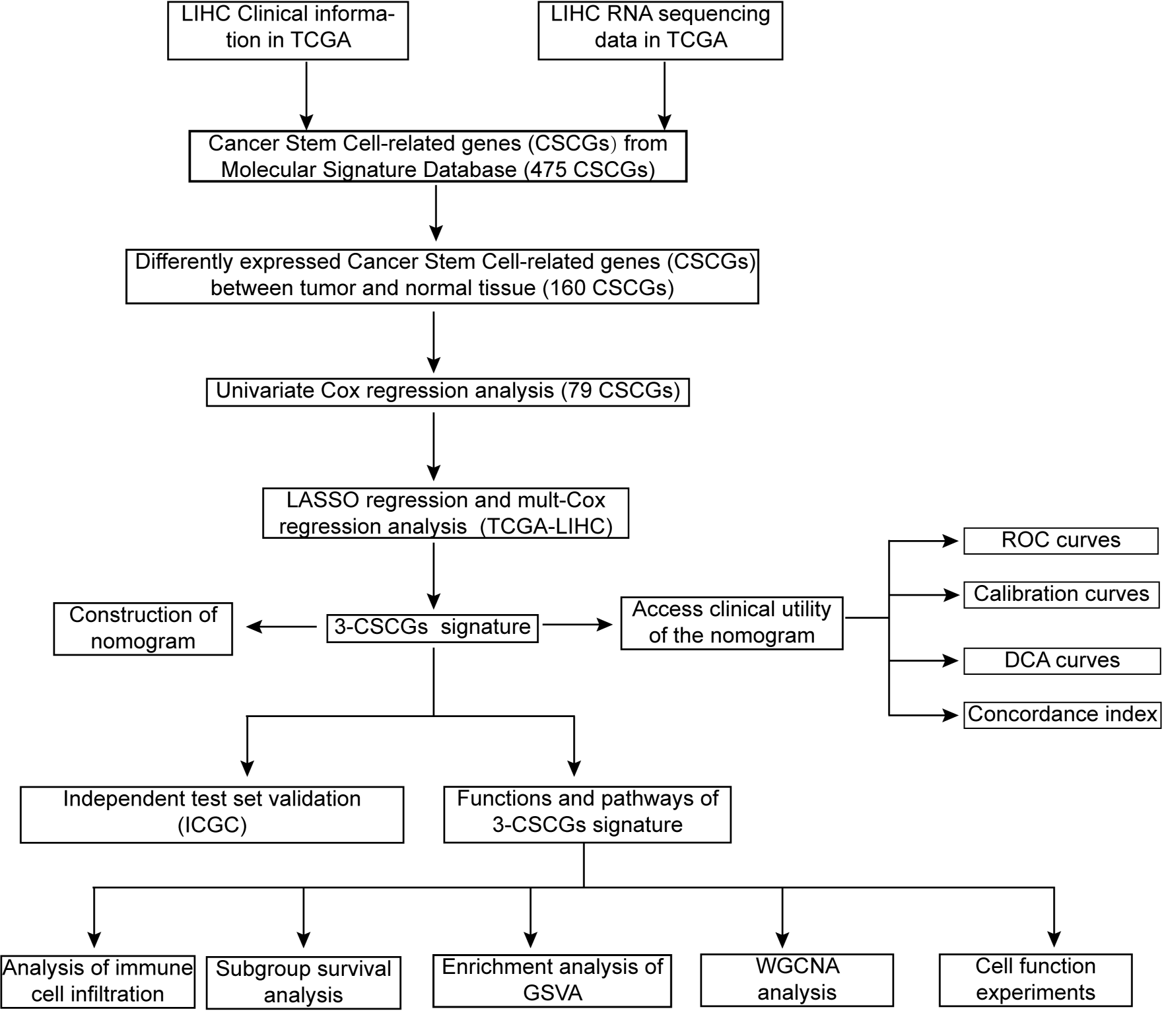

Supplement: Supplementary file 5 [file Image1.TIF]

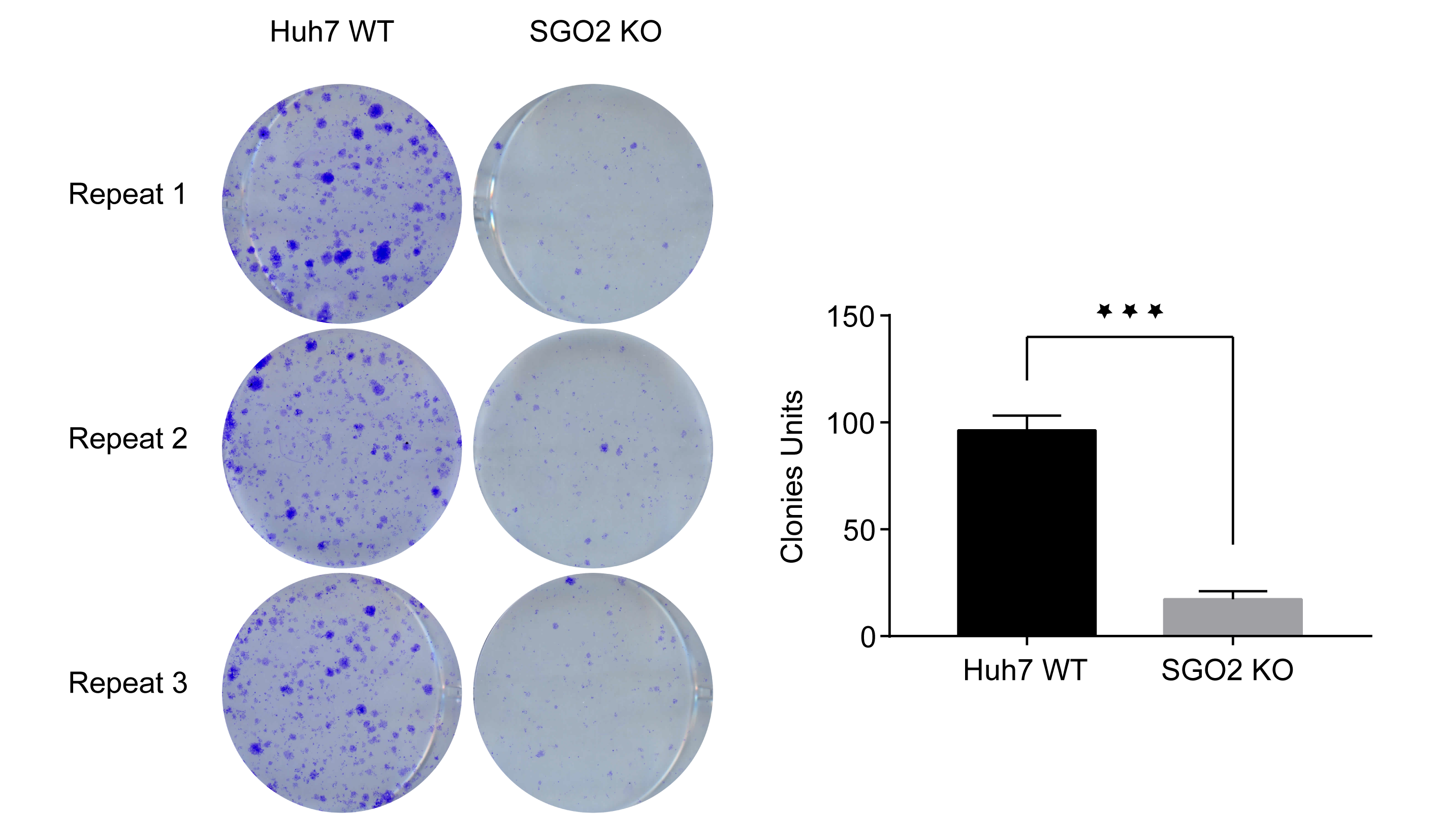

Supplement: Supplementary file 6 [file Image5.TIF]
